# Alterations in sensory and motor nerve conduction associated with age and osteoarthritis in cats are correlated with functional impairment and somatosensory sensitization

**DOI:** 10.1038/s41598-026-60879-y

**Published:** 2026-07-21

**Authors:** Aude Castel, Aliénor Delsart, Colombe Otis, Manuela Lefort-Holguin, Karol-Ann Henry, Maxim Moreau, Bertrand Lussier, Johanne Martel-Pelletier, Jean-Pierre Pelletier, Eric Troncy

**Affiliations:** 1 Groupe de Recherche en Pharmacologie Animale du Québec (GREPAQ), Université de Montréal, Saint-Hyacinthe, QC Canada; 2 Department of Clinical Sciences, Faculté de Médecine Vétérinaire, Université de Montréal, Saint-Hyacinthe, QC Canada; 3 Osteoarthritis Research Unit, University of Montreal Hospital Research Centre (CRCHUM), Montreal, QC Canada

**Keywords:** Electrodiagnostic, Distal neuropathy, Paw withdrawal threshold, von Frey, Feline, MI-CAT(V), Diseases, Medical research, Neurology, Neuroscience

## Abstract

Feline somatosensory impairments associated with aging and osteoarthritis (OA) can be quantified through quantitative sensory testing and nerve conduction (NC). This prospective, randomized and blinded study aimed to compare NC in young healthy (CTRL, *n* = 6) and older OA (OLD, *n* = 12) cats. Under temperature-controlled general anesthesia, amplitude and conduction velocity (CV) of mixed tibial/sciatic and ulnar nerves were measured. Structural changes (radiographic score), functional alterations [MI-CAT(V)], neuro-sensitization [paw withdrawal threshold (PWT); and response to mechanical temporal summation (RMTS)] were assessed. Data distribution determined tests for inter-group comparison, and outcome correlations (α = 5%). OLD cats presented higher radiographic and MI-CAT(V) scores, but lower PWT, RMTS, motor CV (–23%), motor (–35%) and sensory (–45%) amplitudes compared to CTRL. Functional alterations correlated strongly with structural changes (*r* = 0.74, *p* < 0.001) and age (*r* = 0.90, *p* < 0.001), and moderately with neuro-sensitization (*p* < 0.016). Radiographic and MI-CAT(V) scores correlated negatively with (distal) motor CVs (*p* < 0.044), and with some motor and sensory amplitudes (*p* < 0.028). The PWTs correlated positively to motor CVs (*p* < 0.045). Older OA cats exhibited NC alterations correlated with neuro-sensitization and higher than expected with age alone.

**Supplementary Information:**

The online version contains supplementary material available at https://doi.org/10.1038/s41598-026-60879-y.

## Introduction

Age related alteration in mobility is likely secondary to multiple contributing factors involving the joints, the muscles, tendons and ligaments, the nerves and the central nervous system.

In humans, aging is known to be associated with sarcopenia, the progressive loss of muscle mass, function and strength caused by the decline in the quantity and size of muscle fibers and their ability to activate motor units^1^. Age related alterations in the peripheral nervous system have been demonstrated with a gradual decline in nerve conduction velocity (CV) (by 10%) and amplitude (by 40%) in individuals over 60 years (yrs) old^2^.

Osteoarthritis (OA), one of the most common causes of chronic pain, contributes significantly to disability in people^3^. In a study including hundreds of patients over 60-yr-old, structural articular damage was associated with peripheral and central sensitization, as well as heightened anxiety^4^.

Cats are popular companion animals that are similarly affected by aging, with association between age, OA related mobility impairment and chronic pain^5–7^. The OA radiographic prevalence was estimated to be 25.6% in cats between 0.2 and 20 yrs^8^. Radiographic signs of OA were reported in 60% of cats aged 6 + yrs, and this rate increased to 90% in cats aged 12 + yrs^9,10^. Nevertheless, little is known about the impact of age and clinical OA on the peripheral nervous system in this species. Filling this knowledge gap is even more important given that radiographic changes do not correlate linearly with neuro-sensitization or pain perception^11,12^.

Quantitative sensory testing (QST) combines different tests that allow the assessment of somatosensory perception to detect hypo- or hypersensitization to stimuli (including mechanical and thermal ones) in different species including humans, rodents, dogs and cats^13^. These tests have been used to study OA consequences on the nervous system and pain (gain/loss) perception, particularly neuro-sensitization with the imbalance between facilitatory/inhibitory pathways^14–19^.

Indeed, feline OA is known to be associated with functional impairment and QST alterations indicating hypersensitization^5,17,20,21^. More precisely, over the last decade, several methods have been developed and validated to better characterize feline OA pain. Among them, the Montreal Instrument for Cat Arthritis Testing, for use by Veterinarians [MI-CAT(V)], a standardized clinical metrology instrument assessing OA-related functional alterations, and QST have been shown to discriminate between OA and healthy cats (specificity), respond to therapeutics (sensitivity) and be reliable (reproducibility and repeatability)^5,6,17,22,23^. Feline OA occurrence was shown to be highly correlated with age, and OA cats were shown to have significantly lower tactile threshold (i.e., lower paw withdrawal threshold (PWT) using von Frey esthesiometer, characteristic of peripheral sensitization) as well as facilitated temporal summation of pain (characteristic of central sensitization)^17^, two features also identified in human OA^24^. Nevertheless, the underlying peripheral and central nervous system mechanisms leading to feline OA associated hypersensitivity measured with QST remain poorly understood. Lesions involving the nerves were reported in humans with OA as well as in rodent OA models using electrophysiological measurement^25–27^. A recent study in declawed OA cats reported a decrease in tibial/sciatic compound muscle action potential (CMAP) amplitude compared to non-declawed OA cats, but without comparison to healthy cats^28^.

In the 80’s, motor and sensory nerve conduction (NC) parameters were reported in cats, but the drugs used for anesthesia (thiopental, halothane and methoxyflurane) are no longer routinely used (available). Moreover, there was no indication on the neurological and orthopedic status of some of these cats^29,30^. Nerve CV were shown to be stable until about 8 yrs, after which a slow decrease in CV was noted in older cats (average of 25% decrease in 14 year-old cats compared to adult cats under 8 yrs). However, a very small number of cats older than 8 yrs (*n* = 4) were assessed and no correlations were made between the changes reported, as well as functional (OA? ) and sensory impairments^31^.

The objectives of this study were to evaluate and compare NC in motor and sensory nerves of older cats with OA (OLD) and healthy controls (CTRL); and, to assess any correlation between NC variables, age, OA associated structural (radiographic, Rx) and functional [MI-CAT(V)] changes, and neuro-sensitization (QST) values. We hypothesized that NC would be altered in OLD cats compared to younger healthy CTRL confirming some degree of neuropathy, and that the severity of alteration would be correlated with age, and neuro-sensitization, as well as markers of OA functional alteration, but not necessarily with Rx alterations. These results will suggest that OA is not only a joint disease but also a neuropathic disease.

## Results

Descriptive data are presented in Table 1.

**Table 1:** Descriptive statistics representing age, sex, Rx score, MI-CAT(V) score and QST values between both groups. Variables were described as median [min–max], excepted for sex where the number of individuals was included.

Variables	CTRL (*n* = 6)	OLD (*n* = 12)	*p* value
Age (yrs)	1.5 [1.0–4.0]	11.5 [10.0–16.0]	**< 0.001**
Sex	2 F 4 M	7 F 5 M	0.620
Rx (score)	0.0 [0.0–0.0]	10.0 [4.0–19.0]	N/A
MI-CAT(V) score (%)	3.6 [0.0–5.5]	26.4 [20.0–42.7]	**< 0.001**
PWT-thoracic limbs (g)	135.9 [83.8–196.5]	62.3 [24.0–148.1]	**0.001**
PWT-pelvic limbs (g)	144.1 [118.8–199.4]	77.8 [48.0–138.2]	**0.002**
RMTS score (number of stimulation)	26.5 [9.0–30.0]	9.5 [5.0–22.0]	**0.009**

min: minimum value; max: maximum value; yrs: years; Rx: radiographic; MI-CAT(V): Montreal instrument for cat arthritis testing, for use by veterinarians; PWT: paw withdrawal threshold; RMTS: response to mechanical temporal summation; CTRL: young healthy cats; OLD: older and osteoarthritic cats; N/A: non-available.

Significant values are in bold.

As expected, there was a significant difference in age (*p* < 0.001) between CTRL and OLD groups, but no difference in sex-distribution. Additionally, there was a significant difference in the MI-CAT(V) score (+ 633.3%; *p* < 0.001), PWT (–54.2% for thoracic and − 46.0% for pelvic limbs; *p* < 0.002) and the response to mechanical temporal summation (RMTS) values (–64.2%; *p* = 0.009) between both groups. The comparison for the Rx score was not possible considering the Rx value to be null in the CTRL group.

### Comparison between OLD and CTRL cats

Results are summarized in Table 2.

**Table 2:** Mean (and standard deviation) for the different nerve conduction variables measured and calculated for the healthy CTRL and the OLD cats. The relative percentage of variation between CTRL and OLD cats as well as the *p* value resulting from the comparison between both groups are also indicated.

Variables	CTRL	OLD	% of variation (OLD vs. CTRL)	*p* value
	Mean (SD)	Mean (SD)		
**Tibial/sciatic motor nerve**				
Amp tarsus (mV)	36.1 (12.6)	24.1 (12.1)	–33.2	0.067
Amp stifle (mV)	32.5 (11.8)	21.0 (9.7)	–35.4	**0.041**
Amp hip (mV)	28.1 (7.4)	18.9 (8.9)	–32.7	**0.045**
CV stifle-tarsus (m·s^− 1^)	103.0 (11.8)	80.0 (14.8)	–22.3	**0.004**
CV hip-stifle (m·s^− 1^)	108.7 (15.1)	119.4 (29.5)	9.8	0.421
CV hip-tarsus (m·s^− 1^)	105.8 (5.5)	93.8 (13.1)	–11.4	**0.048**
**Ulnar motor nerve**				
Amp carpus (mV)	35.1 (7.8)	21.5 (10.4)	–38.6	**0.013**
Amp elbow (mV)	32.4 (4.8)	22.8 (9.9)	–29.6	**0.014**
Amp axilla (mV)	21.5 (7.1)	20.9 (10.0)	–2.8	0.910
CV elbow-carpus (m·s^− 1^)	105.9 (12.0)	80.3 (14.5)	–24.2	**0.002**
CV axilla-elbow (m·s^− 1^)	124.5 (50.2)	89.2 (26.7)	–28.4	0.066
CV axilla-carpus (m·s^− 1^)	111.6 (22.5)	85.8 (16.5)	–23.1	**0.014**
**Sensory nerves**				
Amp tibial (µV)	28.5 (15.9)	12.4 (5.1)	–56.3	**0.007**
CV tibial (m·s^− 1^)	56.8 (4.5)	55.5 (12.3)	–2.3	0.426
Amp ulnar (µV)	13.6 (5.0)	8.5 (3.8)	–37.5	**0.040**
CV ulnar (m·s^− 1^)	70.1 (13.4)	69.9 (14.7)	–0.2	0.981

CTRL: young healthy cats; OLD: older and osteoarthritic cats; Amp: amplitude; CV: conduction velocity; SD: standard deviation.

Significant values are in bold.

The CMAP amplitude was significantly lower in the OLD cats at the level of the stifle (–35.4%, *p* = 0.041), hip (–32.7%, *p* = 0.045), carpus (–38.6%, *p* = 0.013) and elbow (–29.6%, *p =* 0.014). The sensory nerve action potential (SNAP) amplitude was also lower for both sensory nerves tested (tibial: − 56.3%, *p* = 0.007; and ulnar: − 37.5%, *p* = 0.040). The percentage of decrease of OLD cats relatively to CTRL was approximately 35% for the motor nerves but reached a 47% decrease for the sensory nerves’ amplitude.

The motor CV was also decreased in the OLD group distally for the tibial/sciatic (–22.3%, *p* = 0.004) and ulnar motor nerves (–24.2%, *p* = 0.002) and along both motor nerves tested (tibial/sciatic: − 11.4%, *p* = 0.048 and ulnar: − 23.1%, *p* = 0.014). The percentage of CV decrease was around 23% for the distal CV between CTRL and OLD cats. There was no significant difference in the sensory CV (*p* > 0.426).

When comparing the percentage of variation between OLD and CTRL cats, the most significant alteration was for the SNAP amplitude of the tibial/sciatic nerve, followed by the distal CMAP amplitudes of both nerves and then the SNAP amplitude of the ulnar nerve.

If none of the cases evaluated showed any polyphasia, some OLD cats showed either biphasic or triphasic CMAP of smaller amplitudes which appeared to be slightly more frequent in the thoracic limbs (*n* = 6 cats in the pelvic limbs and *n* = 7 in the thoracic limbs).

### Correlations between NC variables and feline pain phenotype

Correlation between the different NC variables and age, Rx score, MI-CAT(V) score and QST values are summarized in Table 3 and S1.

**Table 3:** Correlation matrix between age, Rx score, MI-CAT(V) and QST values.

	Age		Rx score		MI-CAT(V)		PWT_thoracic		PWT_pelvic	
	Coef	*p* value	Coef	*p* value	Coef	*p* value	Coef	*p* value	Coef	*p* value
Rx score	0.84	**< 0.001**	N/A	N/A	N/A	N/A	N/A	N/A	N/A	N/A
MI-CAT(V)	0.90	**< 0.001**	0.74	**< 0.001**	N/A	N/A	N/A	N/A	N/A	N/A
PWT_thoracic	–0.73	**< 0.001**	–0.66	**0.003**	–0.74	**< 0.001**	N/A	N/A	N/A	N/A
PWT_pelvic	–0.63	**0.005**	–0.45	0.061	–0.58	**0.011**	0.68	**0.002**	N/A	N/A
RMTS	–0.51	**0.031**	–0.57	**0.013**	–0.58	**0.016**	0.50	**0.035**	0.64	**0.005**

Coef: correlation coefficient (*r* for all correlations); Rx: radiographic; MI-CAT(V): Montreal instrument for cat arthritis testing for use by veterinarians; PWT: paw withdrawal threshold

A strong positive correlation was observed between MI-CAT(V) and age, or Rx score, indicating the functional impairments related to OA [MI-CAT(V)] to increase with age (*r* = 0.90, *p* < 0.001) and/or the structural alterations (*r* = 0.74, *p* < 0.001). Interestingly, the functional impairments worsened with both peripheral and spinal sensitization, and the association was stronger for PWT_thoracic (*r* = − 0.74, *p* < 0.001) when PWT_pelvic and RMTS were less sensitive to changes. Indeed, PWT_thoracic was moderately to strongly correlated to all other variables (Table 3).

As expected, a strong positive correlation was observed between the structural alterations (Rx score) and age (*r* = 0.84, *p* < 0.001), which was accompanied by a worsening in functional alterations (MI-CAT(V)) and in neuro-sensitization (PWT_thoracic and RMTS).

### Correlation of NC with age

**Fig. 1:**
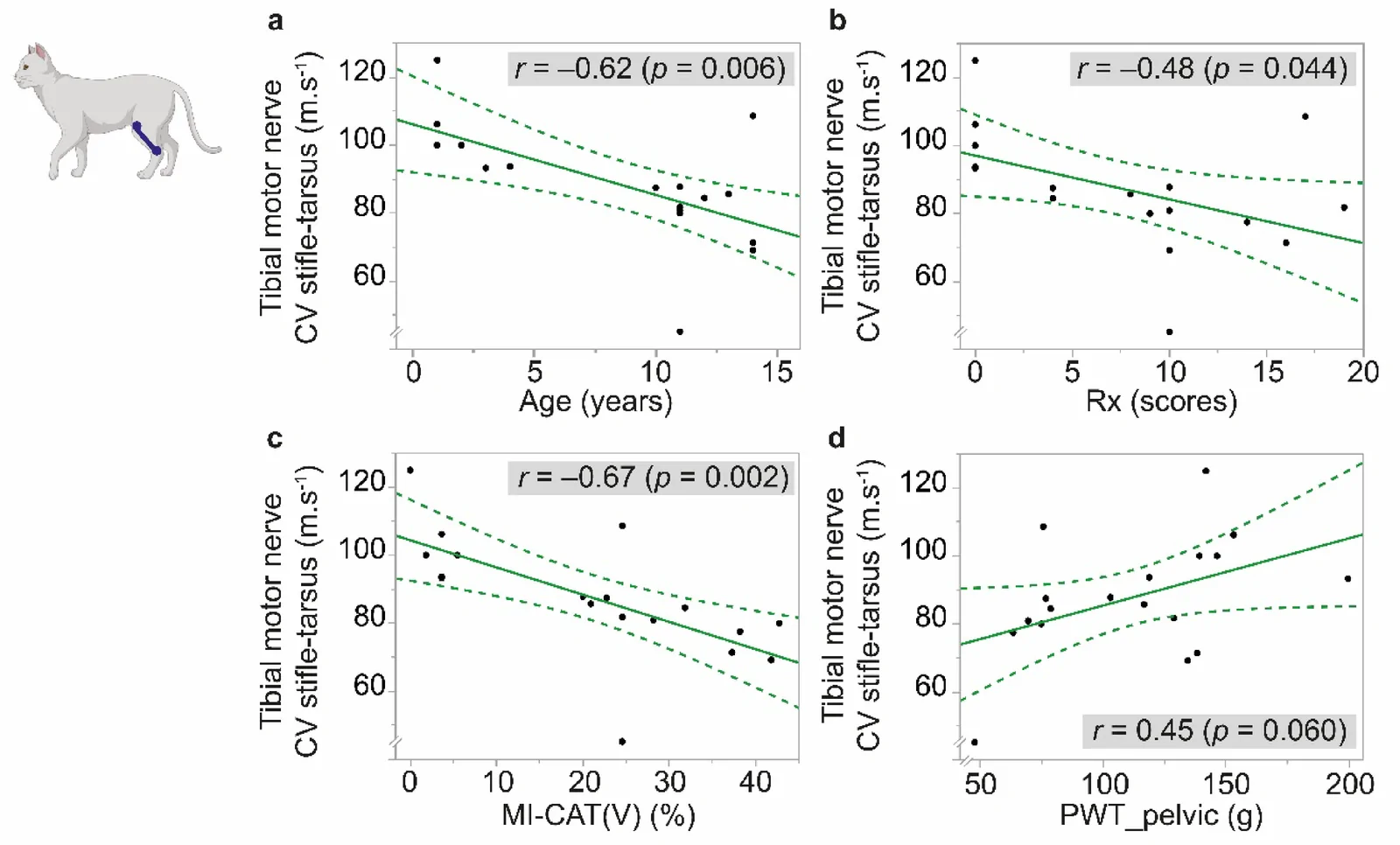
Correlations between distal CV (in m s^-1^) for the tibial/sciatic nerve and (**a**) age, (**b**) Rx scores, (**c**) MI-CAT(V), and (**d**) PWT_pelvic. Linear regression is represented in solid lines and the confidence interval of the fit (at 95%) in dotted lines. CV: conduction velocity; Rx: radiographic; MI-CAT(V): Montreal instrument for cat arthritis testing for use by veterinarians; PWT: paw withdrawal threshold. Created in BioRender. Castel, A. (2026) https://BioRender.com/3d4dvpm.

**Fig. 2:**
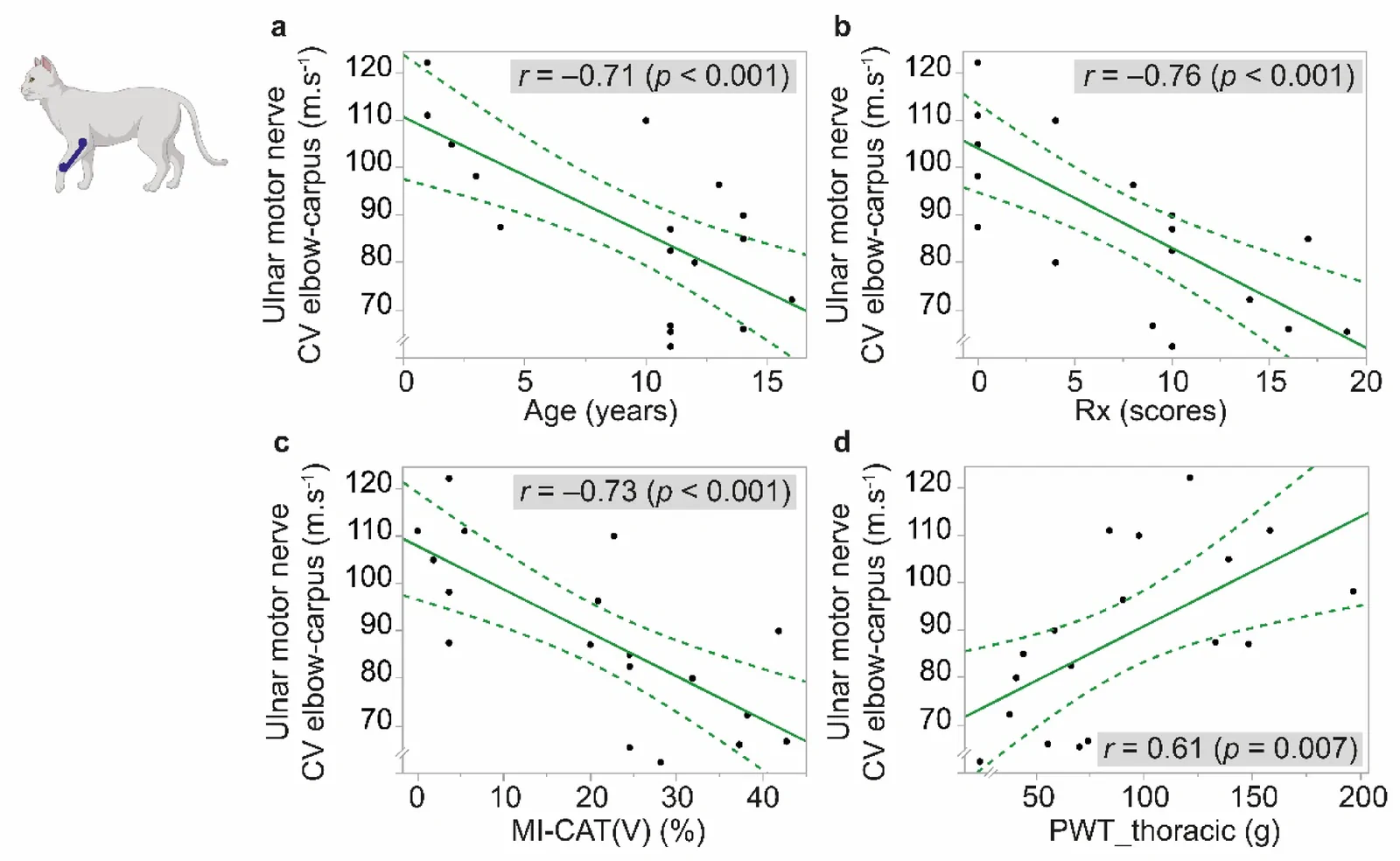
Correlations between distal CV (in m·s^-1^ for the ulnar nerve and (**a**) age, (**b**) Rx scores, (**c**) MI-CAT(V) and (**d**) PWT_thoracic. Linear regression is represented in solid line and the confidence interval of the fit (at 95%) in dotted lines. CV: conduction velocity; Rx: radiographic; MI-CAT(V): Montreal instrument for cat arthritis testing for use by veterinarians; PWT: paw withdrawal threshold. Created in BioRender. Castel, A. (2026) https://BioRender.com/euluvcg.

The CMAP and SNAP amplitudes were moderately negatively correlated with age (Table S1). Motor distal and full nerve CV were also correlated with age (Figs. 1 and 2) for both nerves, with a stronger association for the ulnar nerve. No correlation was observed for the sensory CV.

### Correlation of NC with Rx score

**Fig. 3:**
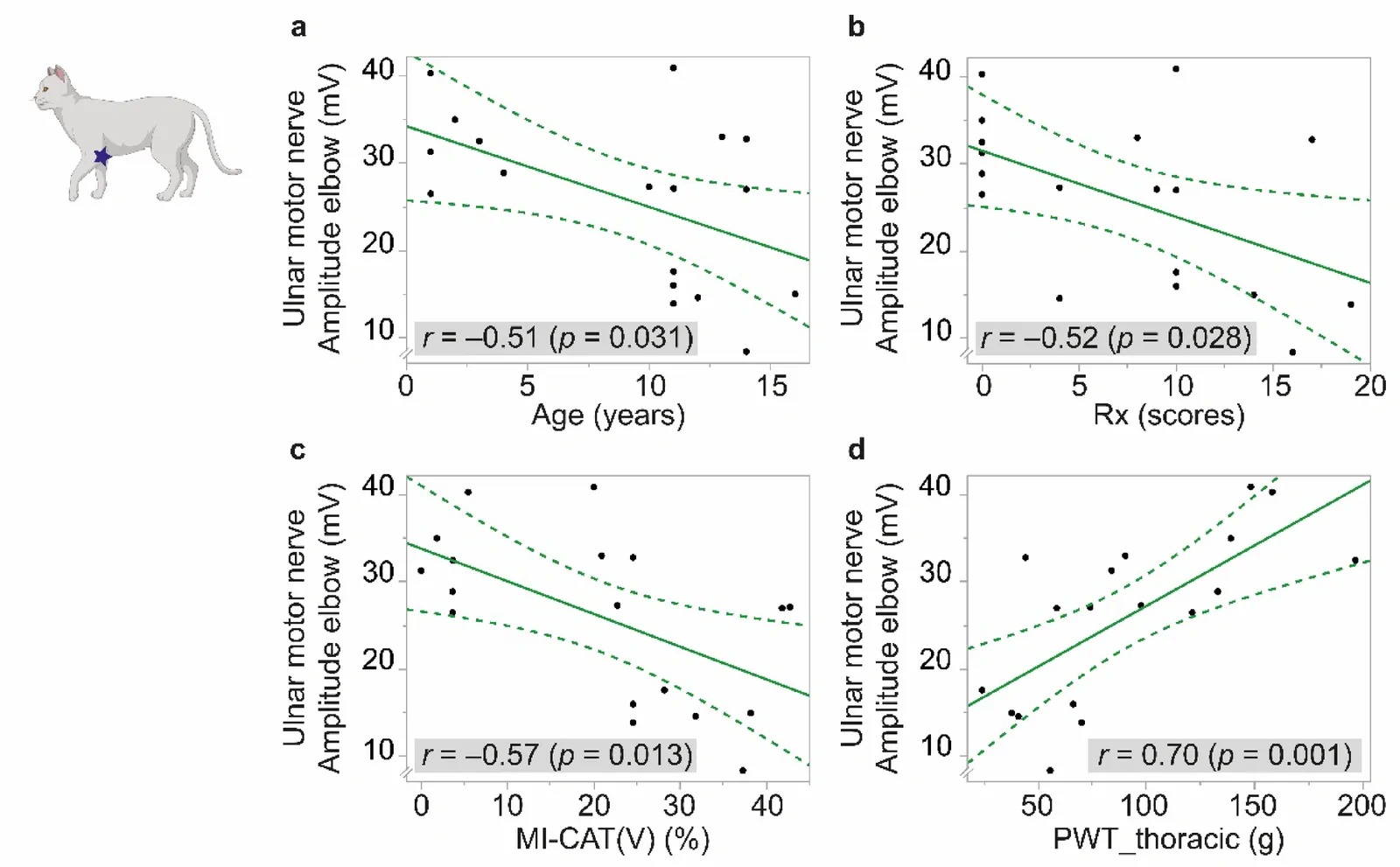
Correlations between the compound motor action potential (CMAP) amplitude at the elbow (in mV) for the ulnar nerve and (**a**) age, (**b**) Rx scores, (**c**) MI-CAT(V), and (**d**) PWT_thoracic. Linear regression is represented in solid line and the confidence interval of the fit (at 95%) in dotted lines. CV: conduction velocity; Rx: radiographic; MI-CAT(V): Montreal instrument for cat arthritis testing for use by veterinarians; PWT: paw withdrawal threshold. Created in BioRender. Castel, A. (2026) https://BioRender.com/55z7tfi.

The Rx score was moderately (mostly) negatively correlated with motor CV distally (tibial/sciatic: *r* = − 0.48, *p* = 0.044; ulnar: *r* = − 0.76, *p* < 0.001) and along the full length of the nerves tested (tibial/sciatic: *r* = − 0.52, *p* = 0.028; ulnar: *r* = − 0.49, *p* = 0.041*)*, with the CMAP amplitude at the elbow (*r* = − 0.52, *p* = 0.028*)* (Figs. 1, 2 and 3) and the SNAP amplitude of the tibial/sciatic nerve (ρ = − 0.57, *p* = 0.018*)*. Cats with a higher Rx score, indicating more severe OA lesions, were associated with more alteration in NC.

### Correlation of NC with MI-CAT(V)

There was a negative correlation between the MI-CAT(V) score and the distal CMAP amplitudes at the tarsus (*r* = − 0.56, *p* = 0.016) and elbow (*r* = − 0.57, *p* = 0.013) (Fig. 3) as well as distal (tibial: *r* = − 0.67, *p* = 0.002; ulnar: *r* = − 0.73, *p* < 0.001) and full-length (tibial/sciatic: *r* = − 0.55, *p* = 0.019; ulnar: *r* = − 0.64, *p* = 0.004;) CVs for both nerves. No correlations were found for sensory amplitude or CVs. This indicates that OA-related functional impairments increases with the motor nerve alterations.

### Correlation of NC with PWT

The PWT of the thoracic limbs was strongly positively correlated with the CMAP ulnar amplitude measured at the level of the elbow (*r* = 0.70, *p* = 0.001) (Fig. 3).The PWT_thoracic was also positively correlated to the motor CV (distally i.e., between the elbow and the carpus) (*r* = 0.61, *p* = 0.007), proximally i.e., between the axilla and the elbow (*r* = 0.68, *p* = 0.002) and for the full length of the ulnar nerve (*r* = 0.67, *p* = 0.002). Interestingly, PWT_thoracic was moderately associated to one NC variable measured on hind limb, only.

The PWT of the pelvic limbs presented a moderate positive correlation with one NC variable, i.e. the CV for the full length of the tibial/sciatic nerve (*r* = 0.48, *p* = 0.045), confirming its lower sensitivity. There was no correlation between PWT_pelvic and the SNAP amplitude for both nerves tested. In final, this suggests the peripheral neuro-sensitization to be mostly associated with a reduced CV.

### Correlation of NC with RMTS

There was no correlation between the RMTS values and any of the NC variables assessed for both motor and sensory portions of the two nerves tested. This confirms this dynamic QST to assess spinal sensitization, independently of peripheral nerves function.

## Discussion

Confirming our hypothesis, NC was altered in OLD cats affected by OA compared with younger healthy CTRL cats with the most pronounced decrease affecting the amplitudes of the motor and sensory nerves tested (–35% to − 45%). As expected, age and Rx score were correlated confirming the amplification of structural alterations assessed through X-rays with aging^9,10,32–34^. The observed strong positive correlation between MI-CAT(V) assessing the degree of OA-associated functional impairments and both age and Rx score confirmed the validity (sensitivity) of the MI-CAT(V) clinical metrology instrument. The functional impairments aggravated too with an increase in neuro-sensitization, either peripheral (PWT), or central, spinal (RMTS). The inverse relationship (i.e., the increase in functional impairments alters the neuro-sensitization) is possible. The decrease in PWT, indicating tactile sensitization, was associated with a reduction in CV (or the inverse), but the spinal RMTS was not sensitive to appendicular nerves alteration. Therefore, these findings support the hypothesis that OA chronic pain is associated with neuro-sensitization, both tactile and spinal, that worsens with aging and structural alterations. The neuro-sensitization and the OA condition are associated with nerve function (amplitude and CV) alterations with cats presenting the slowest motor CVs showing a lower mechanical threshold (PWT) indicating tactile sensitization.

In dogs, nerve CV of the tibial/sciatic were reported to remain constant between 1- and 7-yr-old, followed by a 10 to 15% reduction by 10 yrs of age^35^. In humans, nerve CV was reported to decrease by 10% between the age of 30 and 60 yrs^36^, as well as by 10% in nerve CV and by 40% in amplitude for individuals over 60 yrs^2^. The decrease in OLD cats motor nerve CVs, particularly in the distal segments, was more important (–23% in average) than what has been reported in the literature. This suggests that the OA neuropathology aggravated the reduction in CV. In cats, nerve CV in group I fibers in the gastrocnemius medialis nerve and lumbar alpha motor neurons were slower in 14- and 15-yr-old cats than in cats aged between 1- and 3-yr-old^37,38^. In Morales, et al. ^39^, slower motor CV was reported but it is difficult to compare the results with ours because pentobarbital anesthesia and a very invasive technique were used as the nerves were excised and positioned on the stimulating electrodes. In another cat study by Pillai, et al.^31^, motor and sensory CVs were shown to remain constant between 2 and 8 yrs. One 9- and 10-yr-old cats presented CV within two standard deviations of the overall means for 1- to 8-yr-old cats, and the mean CV for the two 14-yr-old cats was 30% slower for the sensory and approximately 20% for the motor nerves compared with cats between 1 and 8 yrs. The SNAP and CMAP amplitudes were not significantly different with age in their population, suggesting that changes in amplitude were more specific to OA than age in our study. However, in the older cats’ group of their study, only 4 individuals were included with significant variability, and they were compared with another group of cats from 1- to 8-yrs old with also significant variability between individuals attested by the high standard deviations and high variability from one year to the next. Cats in this previous study were not submitted to Rx evaluation; therefore, the presence of chronic pain could not be excluded to explain these variabilities. Furthermore, they only looked at correlation with age as a linear progression but did not compare younger adults to older ones. Studies in rodents have found that age-related changes in the nervous system were not linear^40^. It is also possible that age-related changes causing alterations in feline NC start after the age of 10 yrs but because only 2 individuals above this age were included, inferential statistics were impossible.

In contrast with the study by Pillai et al.^31^, where CVs remained constant until 8 yrs, a moderate negative correlation was found between age and most of the NC variables. The strongest correlation with age was found for the distal ulnar motor nerve CV. However, the higher percentage of variation between CTRL and OLD cats was for the SNAP amplitude of the ulnar and tibial/sciatic nerves showing a 38% and 56% decrease respectively. A similar result was reported in humans affected by hand OA with a halved SNAP amplitude compared to healthy individuals with the same age range, suggesting axonopathy and loss of nerve fibers functionality^41^. In our study, age and chronic pain were correlated, and in elderly humans a SNAP amplitude decrease was also the most significant change when comparing with young subjects^2^. Interestingly, in human as well, the rate of decline was not necessarily linear but tended to be better predicted by a quadratic parabolic model^2^. Interestingly, in the elderly individuals, sensory symptoms in the legs (pins and needles, cramps) were also increasing with age but this type of symptoms is difficult to assess in non-human animals.

Another factor of variability to take into consideration when comparing the different NC studies is the effect of the anesthesia protocol. Indeed, in some animal studies the excitability of primary sensory afferents was significantly reduced by propofol and midazolam in-vitro^42^,^43^. In rodents, ketamine and pentobarbital were associated with a significantly lower motor sciatic CV, but no change in sensory CV was observed compared to isoflurane^44^. In humans, propofol was found to have minimal to no impact on peripheral NC^45,46^. In the present study, anesthesia was maintained with a propofol CRI with the goal to minimize hypotension, as isoflurane tends to cause more pronounced vasodilatation and decrease in body temperature and in blood pressure that could have impacted the kidneys of OLD cats^47^. Another study in dogs had also shown that hypothermia was more pronounced with isoflurane compared to propofol which could impact NC^48–50^. In accordance to a pilot study, fentanyl was administrated for analgesia as this opioid had the least effect on body temperature compared to methadone and hydromorphone, optimizing the NC studies^51^.

The MI-CAT(V) scale was used to identify functional impairment affecting the body posture, gait, and ability to pass over and under obstacles in the cats evaluated. As expected, there was a correlation between age and both Rx and MI-CAT(V) scores with OLD cats having higher scores indicating higher structural and functional impairments. The OLD cats assessed in this study were from a colony of cats with various degree of OA and were known to have some mobility impairment associated with the condition. Interestingly, the severity of functional impairment was not only correlated with age and Rx score, but also with alteration in motor NC and concomitant aggravation in neuro-sensitization. This is in line with a previous study showing declawed cats were more affected by functional OA alterations than non-declawed cats^28^.These results are interesting because, to our knowledge, no study in cats has assessed gait changes associated with NC alterations with objective methods such as gait analysis (force plate analysis among others) or functional scales including both healthy and cats affected by chronic pain. Therefore, these results confirm the validity (sensitivity) of MI-CAT(V), and even if the MI-CAT(V) scale was initially developed to assess OA cats^6,22,23,52^, it could be also useful to assess more objectively cats with neuropathy and their response to treatment. With the present results, one can wonder whether the functional impairments result from age-associated nerve lesions and/or are consecutive to some neural degeneration induced by the OA neuropathological processes (neuro-inflammation, centralized sensitization, etc.). One potential explanation could be that age associated loss of large myelinated fibers alter the protective effects of the proprioceptive reflex necessary to stabilize the joints which could render the joints more susceptible to abnormal loading and accelerate their deterioration, as suggested by McDougall^53^. Interestingly, thinning of nerve fibers in the joint was also reported in horses with severe cartilage damage from OA, suggesting that equine OA could also be associated with peripheral neuropathy^54^.

The decreased CMAP and SNAP amplitudes, as measured in the OLD cats, are usually suggestive of motor and sensory axonal damage, respectively, but can be seen with severe demyelinating diseases or conduction block^55^. Additionally, motor CV was slower in OLD cats, but not sensory CV. Slower nerve CV is usually associated with myelin loss, but can be in part due to axonal loss, particularly when the largest and fastest conducting fibers are affected^55^. These results could suggest that age or OA-associated myelin loss mainly affects the motor fibers but spare the sensory fibers. In the present study, without histopathological confirmation, it is difficult to know whether the NC alterations observed result from axonal loss only or from a combination of axonopathy and demyelination and whether OA associated neuro-pathological processes could trigger these changes. In a previous study, segmental demyelination with minimal axonal degeneration affecting the large and medium diameter fibers was reported in old cats over 14 yrs of age (*n* = 2), which was also associated with slower CV^56^. Interestingly, segmental demyelination and remyelination as well as Wallerian degeneration followed by regeneration were reported in aged humans and dogs^57,58^. Unfortunately, the possible relationship between OA-associated neuropathological processes and nerve changes has not yet been examined. Nevertheless, a study in horses with OA reported a reduction in synovial nerve fiber density, where the decrease in density in superficial nerve fibers was associated with OA severity but not with age, suggesting a link between OA and nerve damage in this species^54^. In feline OA, conducting histopathological assessments of nerve fibers and evaluating the potential correlations with OA severity would be pertinent to confirm whether OA constitutes a neuropathic disease. Notwithstanding this limitation, these results present, to the best of our knowledge, the first evidence that feline OA is associated with axonopathy, reduced nerve fiber functionality, and myelin loss, that appear more pronounced than changes attributable to aging alone.

Neuro-sensitization was associated with lower CMAP amplitude, as previously reported in a smaller cohort of OA declawed cats^28^. Interestingly, increase in neuro-sensitization was mostly associated with decreased CVs along ulnar and tibial nerves, when it was expected that the PWT would increases as the cat would take more time to withdraw the paw in response to the stimulation (due to the reduced CV). Tactile stimulation assesses the function of the large, myelinated fibers which is also the case for NC^59^. In humans, ageing is associated with increased tactile thresholds^60^. Conversely, humans with OA pain had lower mechanical threshold than healthy controls^61^. Interestingly, in one study, almost 30% of individuals with knee OA had symptoms suggestive of neuropathic pain at the affected joint^62^. Similarly, PWT and RMTS score were shown to be lower in cats with OA, indicating OA-associated peripheral and central sensitization in this species^17^. Therefore, the lower PWT in the OLD population suggests that these cats had peripheral sensitization and that the slower motor CV is not clinically significant enough to impact the paw withdrawal latency in these sensitized animals. Indeed, the sensory CV was not impacted, and peripheral sensitization of the remaining functional nerve fibers may have driven the observed reduction in PWT.

The lack of correlation between the RMTS and NC alteration was not surprising as it is suspected that RMTS assesses central sensitization, mainly at the level of the spinal cord^21^, whereas NC evaluates the peripheral nervous system. However, the lower RMTS score of OA cats along with lower PWT and alteration in both sensory and motor NC, suggest that OLD cats with OA likely suffer from a combination of both central and peripheral sensitization. This dual involvement points to multiple different therapeutic targets that could be combined to optimize the treatment response.

It is important to discuss the limitations of the study. All the OLD cats had pelvic limbs Rx lesions compatible with OA. It would have been ideal to have included cats with only axial lesions to our population, as well as cats without any OA lesion. To validate the NC changes observed in this study, future investigations should include a larger sample size consisting of a control group (without OA) age-matched with the OA group, to strengthen the study’s design and further support the conclusions. However, given the high prevalence of this condition, with over 60% of cats older than 6 yrs showing OA associated Rx changes, it would be extremely challenging to find such cases^10^. Another limitation is the lack of histopathological confirmation of the nerve damage suspected with the observed NC alterations. Histopathological evaluation including immunohistochemistry and teased fiber techniques would be useful, especially given that NC test detects changes affecting only the large fibers, whereas small fiber losses are not assessed with this technique despite potentially playing a role in hypersensitivity^63,64^.

Although peripheral nerve histopathological changes associated with age have been described in cats, no correlations were made with electrodiagnostic evaluations and QST results. Given the invasive nature of nerve biopsies and the risk of worsening functional impairment in already compromised and painful cats, the invasiveness of the procedure is difficult to justify for our cat colony despite the important information that it could provide. Magnetic resonance imaging (MRI) can help assess nervous tissue such as the brain and the spinal cord. It would have been interesting to perform the procedure to exclude peripheral and central nervous lesions that could have impacted results presented. However, given that the changes tended to be more severe distally, the resolution of MRI to assess smaller structures like distal nerves of cats would likely be insufficient with regular 1.5 or even 3T magnets and would likely require the use of much stronger magnet. Another electrodiagnostic test, the H-reflex, or F-wave, two tests useful to assess the most proximal motor nerve segments and reported to be altered in elderly humans were not performed^2,65–67^. Further studies are therefore needed to further characterize age-related changes in the cat’s peripheral nervous system and their association with degenerative joint disease and chronic pain.

In conclusion, OLD cats affected by OA exhibited alterations in motor and sensory NC compared to younger healthy cats. Older cats and those with more severe functional impairments, structural damages, and neuro-sensitization presented a decrease in both amplitude and CV in mixed tibial/sciatic and ulnar nerves. This study suggests that OA involves not only the joint(s) but also neuropathic mechanisms, which are likely to contribute to impaired mobility and reduced well-being.

## Materials and methods

### Study design and animals

This prospective, randomized and blinded study was approved by the Institutional Animal Care and Use Committee of ArthroLab inc. (#A196-ART22F) and of the Faculty of veterinary medicine (#CEUA-Rech-2114) of Université de Montréal. Management of animals adhered to the regulations outlined in the Canadian Council on Animal Care, Guide to the Care and Use of Experimental Animals, Vol. 1, 2nd Edition (1993, revised in Feb. 2017), and complied with the US Dept. of Agriculture Animal Welfare Act (9 CFR Parts 1–3). All procedures were also conducted in accordance with the ARRIVE guidelines for reporting animal research.

Six young healthy CTRL adults (less than 4-yr-old) and 12 senior OLD cats (between 10- and 16-yr-old) with OA from a research colony were included (Table 1). All the cats had a physical, orthopedic examination, performed by a board-certified Veterinary Surgeon (B.LU.), and neurologic examination, performed by a board-certified Veterinary Neurologist (A.CA.), as well as blood work (complete blood count and biochemistry including T4 measurement). All the examinations were performed blindly. Exclusion criteria were the presence of neurological deficits or significant sign of health comorbidities (other than OA for the OLD cats, such as advanced renal disease, cardiac disease…). Cats were group-housed in lighting-, temperature-, and humidity-controlled rooms with environmental enrichment (perches, beds, scratching posts and toys). Cats were fed twice daily with veterinary quality commercial foods according to the manufacturer’s recommendations and fresh water was available *ad libitum*.

### Radiographic scoring of structural alterations

Each cat underwent Rx evaluation of the axial skeleton, as well as thoracic (carpus, elbow, shoulder) and pelvic (tarsus, stifle, hip) appendicular joints. A board-certified Veterinary Surgeon (B.LU.) reviewed and scored all X-rays independently and blindly. The Rx score corresponds to the summation of a Likert-type severity scale (0–5, null to most severe OA) of the twelve joints evaluated, added to the number of joint(s) affected by Rx OA. To be included in the OLD group, a cat had to present some Rx alterations (i.e., presence of osteophytes and/or subchondral sclerosis or cyst) in at least one pelvic appendicular joint, excluding those with axial lesions. Lesions such as meniscal mineralization or enthesiophytes had to be associated with osteophytes and/or subchondral alteration to be clinically significant. Conversely, cats were considered healthy CTRL if no OA lesion was present in any of the joints evaluated (score of 0).

### Functional assessment with the MI-CAT(V)

All cats were acclimated to the room and evaluator (E.TR.) for a minimum of three sessions. They were assessed blindly, once for CTRL cats or twice for OLD cats (the median was used for analyses), for functional abilities with the MI-CAT(V) scale previously described and validated (reliable, specific and sensitive to OA pain)^6,22,23,52^. The version used for this study consisted in four standardized sections scored independently: body posture, gait, obstacles, and global distance examination^23^. The scores of each independent section were added for a maximum of 55 to obtain a cat’s total score, expressed in percentage, with higher MI-CAT(V) scores indicating greater impairment in the cat’s functional abilities.

### Quantitative sensory testing

All cats underwent two types of QST: the PWT measuring, in grams (g), punctate tactile sensitivity and predominantly reflecting peripheral sensitization, and the RMTS, a count-based test assessing central sensitization^5,17,21,68,69^. All cats were previously acclimated to the procedure and evaluators (A.D., M.L.H. and two technicians trained to perform the procedures) for a minimum of six sessions. Each assessment was performed blindly, and a reliability number was attributed according to a Likert scale (0: poorly reliable, 1: moderately reliable and 2: highly reliable). One assessment evaluation was conducted for CTRL cats and two (at 14-day interval) for OLD cats (the median was used for subsequent analyses).

### Peripheral sensitization assessment—the PWT

The PWT was evaluated by application of an electronic von Frey esthesiometer to the plantar/palmar paw surface. The von Frey esthesiometer consisted in a polypropylene probe (Rigid Tip 0.7 mm^2^ of surface 28G, IITC Life Science, Woodland Hills, CA, USA) fitted on a hand-held force transducer allowing to apply gradually increasing pressure and to record the maximal pressure tolerated. The tip was placed perpendicularly into the plantar/palmar surface of the four paws while the cat was standing up in a cage with a mesh. The stimulus was applied with increasing pressure until the paw was withdrawn or elevated such that the force leveled off, or until any other reactions such as vocalization, agitation, jumping and avoidance was witnessed. For each animal, a duplicate measurement of each applied paw peak force (g) was taken. A total of eight PWT measurements was obtained, with an approximately 2 min interval between duplicate, in a randomized order (the order changed from one session of four paws to another to avoid cat habituation and stimulus anticipation). Data under 2 g were considered unreliable and discarded, and a maximal cut-off value of 200 g (to avoid any injury) was applied. The lower the threshold, the more the cat is considered peripherally sensitized (with a central participation). The weighted mean using the reliability number was calculated for both PWT_thoracic (4 values for CTRL, 8 for OLD cats) and PWT_pelvic (4 values for CTRL, 8 for OLD cats) for each cat.

### Central sensitization—the RMTS

Repeated mechanical stimuli of sub-threshold intensity (one stimulus does not elicit a pain behavior), at fixed intensity (4 N), frequency (0.38 Hz) and duration (up to 30 stimulations over 80 s) were applied using a purpose-made device (Top Cat^®^, Bespoke Measurement Systems, Cambs, UK)^21,68,69^. Round-ended metallic pin of 2.5 mm diameter, 10 mm long, mounted in a light rolling diaphragm actuator, adapted from a validated mechanical ramped stimulus testing device provided the mechanical stimuli. Inserted in a small band around the limb, the mechanical stimulator was placed on the cranial aspect of the right mid radius/ulna. A ‘dummy’ device was placed on the contralateral limb. During the evaluation, the cats were free to move but restricted in a meshed cage.

During an evaluation, the stimulation set was stopped by the evaluator (A.DE.) when a withdrawal response (e.g., vocalization, agitation, striking the band containing the stimulator, or avoidance) was detected, or stopped automatically when the maximal number of stimuli (30) was reached. Each evaluation included a duplicate measurement: a 4-min interval between both stimulation sets was respected, allowing cats to express normal behavior in a positive enrichment environment. The weighted mean using the reliability number was calculated (2 values for CTRL, 4 for OLD OA cats) and used for subsequent analyses. The lower the number of accepted stimulations, the more the cat was centrally sensitized. The RMTS has been associated with spinal wind-up, facilitating, and amplifying nociceptive signals^13,17,28^.

### Nerve conduction (NC) study on anesthetized cats

#### Anesthesia protocol

This evaluation was conducted several days after MI-CAT(V) and QST to avoid any interference with anesthesia. After being fasted for 4 to 8 h, cats received one subcutaneous dose of 5 µg/kg dexmedetomidine at GV20 site (Dexdomitor^®^ 0.5 mg/mL, Zoetis Canada, Kirkland, QC, Canada) to allow shaving of the forearm and intravenous (IV) catheter placement in the cephalic vein. The anesthesia was induced by IV propofol (3 mg/kg, Baxter^®^ Propofol injectable emulsion vial 10 mg/mL, Baxter Corporation, Mississauga, ON, Canada) then an IV fentanyl bolus (3 µg/kg, Fentanyl Citrate Injection USP^®^ 50 µg/mL, Sandoz Canada, Boucherville, QC, Canada) was administered. The cat was kept on 30–35% enriched oxygen (1.0–1.5 L/min by mask) and fluid therapy (3 mL/kg/h, Lactate Ringer’s Injection USP^®^, Baxter Corporation) throughout the procedure. Fentanyl was selected as the opioid analgesic, as an internal pilot study identified it was the optimal agent for facilitating reliable NC assessment while limiting body-temperature loss when compared to hydromorphone and methadone^51^.

A plane of general anesthesia was maintained with a propofol continuous rate infusion (0.18–0.24 mg/kg/min Syringe Pump DigiPump SR31x^®^, Digicare Biomedical Technology, Boynton Beach, FL, USA). To maintain body temperature above 37.5 °C (99.5 °F), a heating pad (Veterinary Heat Pump Patch^®^ / HTP-1500^®^, Adroit Medical Systems, Loudon, TN, USA) covert and warm bottle were concomitantly used. Continuous anesthesia monitoring (LW6000^®^ Lifewindow™ monitor, Digicare Biomedical Technology, Boynton Beach, FL, USA, and manually) included evaluation of eye reflexes, heart rate and rhythm, oscillometric systemic blood pressure measured through an adapted cuff placed on the tail or on a limb (not used for NC), pulse oximetry on the tongue, respiratory rate, capillary refill time, mucous membrane color and rectal temperature recorded every 5 min. The ETCO_2_ was not monitored during the procedure as the cats were not intubated.

#### NC evaluation

Nerve conduction was performed blindly by a board-certified Veterinary Neurologist (A.CA.) with the Sierra II Wedge/EP^®^ system (Caldwell Industries, Kennewick, WA, USA) on two mixed nerves: the ulnar (left) and the tibial/sciatic (right) nerves using subdermal needle electrodes positioned as previously reported^30,70,71^.

To assess motor NC, stimulating electrodes (cathode and anode) were placed in close proximity of the tibial/sciatic nerve at the level of the tarsus, stifle, and hip and electrical stimulation was applied until it became supramaximal i.e., the maximal amplitude of the CMAP was reached and remained unchanged despite increasing the stimulus intensity. The distance between the different stimulation points was measured with a tape (in cm). Latency (in ms) and amplitude (in mV) of each CMAP obtained at the different sites were recorded and the CVs (in m·s^− 1^) between each segment (stifle-tarsus, hip-stifle and hip-tarsus) were calculated. Similarly, CMAPs were obtained, and latencies and amplitudes recorded at the carpus, elbow and axilla to assess motor NC along the ulnar nerve and CVs were calculated between each segment (elbow-carpus, axilla-elbow and axilla-carpus). The presence of polyphasia which indicates that the CMAP waveform is crossing the baseline five or more times was recorded if present as potential marker of nerve fibers regeneration.

To assess sensory NC, only one site was assessed per nerve tested i.e., the carpus for the ulnar and the tarsus for the tibial/sciatic in an orthodromic direction i.e., with the stimulating electrode in the interosseous muscles of the metacarpus and metatarsus, respectively. The intensity of the electrical stimulus was slowly increased until a SNAP of maximal amplitude was reached. The latency (in ms) and amplitude (in µV) were recorded and the CV (in m·s^− 1^) calculated after measuring the distance between the stimulating and recording electrodes.

### Statistical analysis

The statistical analysis was performed with the software JMP-Pro 17.0.0^®^ (JMP Statistical Discovery, Cary, NC, USA). Correlation graphs were created with this software (in Figs. 1, 2 and 3). Based on a previous study on nerve conduction in cats^31^, we estimated that the standard deviation (SD) for a normal mean velocity for the sensory tibial nerve was 61.3 +/-7.3 m.s^-1^. We considered that a decrease of 16% or more would likely be significant and not just age-related (considering that a 10–15% decrease has been reported in older dogs). Using this information, a sample size calculation with a significance level of 0.05 and a power of 0.80, determined that the sample should consist of 18 individuals. A total of 18 cats (12 OLD and 6 CTRL), were ultimately included (some cats that were supposed to be in the CTRL had developed OA lesions). The relative difference of OLD comparatively to CTRL for NC values was calculated as a percentage of variation using the delta between both groups divided by CTRL value. The Shapiro-Wilk test was performed to assess data distribution followed by the Levene test to evaluate variance homogeneity. Comparison of NC variables (amplitude and CV) between OA and CTRL groups was realized with a Student *t*-test for normally distributed variables and a Wilcoxon test for non-Gaussian distributed variables. Correlations between age, Rx and MI-CAT(V) scores, PWT_thoracic, PWT_pelvic, RMTS values, between them, and with the different NC variables were assessed using Pearson correlation test for normally distributed variables and Spearman rank test for non-Gaussian distributed variables. The coefficient of correlation (*r* for Pearson and ρ for Spearman) was interpreted as followed: 0.00–0.39 = weak, 0.40–0.69 = moderate and 0.70–1.00 = strong agreement^72^. A *p* value < 0.05 was considered statistically significant.

## Supplementary Information

Below is the link to the electronic supplementary material.

Supplementary Material 1

## Data Availability

The datasets presented in this study can be found in an online repository: [https://data.mendeley.com/datasets/mcyf43jcj7/1](https:/data.mendeley.com/datasets/mcyf43jcj7/1).
