# Supplementary material for: Alterations in sensory and motor nerve conduction associated with age and osteoarthritis in cats are correlated with functional impairment and somatosensory sensitization
Source: Sci Rep. 2026 Jul 21;16:33097. doi: 10.1038/s41598-026-60879-y (PMC13683033; doi:10.1038/s41598-026-60879-y)
Supplement: Supplementary file 1 [file 41598_2026_60879_MOESM1_ESM.pdf]

Supplementary material

S1 – Correlations between nerve conduction variables and feline pain phenotype.

	Age (years)		Rx (score)		MI-CAT(V) (%)		PWT_thoracic (grams)		PWT_pelvic (grams)		RMTS (count)	
Variables	Coef	*p* value	Coef	*p* value	Coef	*p* value	Coef	*p* value	Coef	*p* value	Coef	*p* value
**Tibial/sciatic motor nerve**												
Amp tarsus (mV)	**–0.49**	**0.038**	–0.37	0.131	**–0.56**	**0.016**	0.44	0.065	0.39	0.107	0.14	0.578
Amp stifle (mV)	**–0.49**	**0.040**	–0.21	0.407	–0.38	0.119	0.13	0.619	0.30	0.233	0.12	0.639
Amp hip (mV)	**–0.47**	**0.049**	–0.30	0.230	–0.42	0.085	0.28	0.261	0.38	0.122	0.08	0.766
CV stifle-tarsus ($m.s^{-1}$)	**–0.62**	**0.006**	**–0.48**	**0.044**	**–0.67**	**0.002**	0.36	0.138	0.45	0.060	0.28	0.262
CV hip-stifle ($m.s^{-1}$)	0.11	0.664	–0.07	0.786	0.14	0.584	0.07	0.795	0.01	0.982	–0.01	0.995
CV hip-tarsus ($m.s^{-1}$)	**–0.52**	**0.027**	**–0.52**	**0.028**	**–0.55**	**0.019**	**0.50**	**0.034**	**0.48**	**0.045**	0.31	0.216
**Ulnar motor nerve**												
Amp carpus (mV)	–0.46	0.057	–0.36	0.146	–0.39	0.120	0.38	0.119	0.38	0.118	0.11	0.654
Amp elbow (mV)	**–0.51**	**0.031**	**–0.52**	**0.028**	**–0.57**	**0.013**	**0.70**	**0.001**	0.36	0.143	0.23	0.351
Amp axilla (mV)	0.11	0.651	0.14	0.570	–0.04	0.866	–0.01	0.992	0.21	0.412	0.16	0.523
CV elbow-carpus ($m.s^{-1}$)	**–0.71**	**<0.001**	**–0.76**	**<0.001**	**–0.73**	**<0.001**	**0.61**	**0.007**	**0.48**	**0.042**	0.30	0.229
CV axilla-elbow ($m.s^{-1}$)	–0.43	0.073	–0.42	0.081	–0.45	0.063	**0.68**	**0.002**	**0.62**	**0.001**	0.20	0.424

CV axilla-carpus (m.s^{-1})	**–0.60**	**0.008**	**–0.49**	**0.041**	**–0.64**	**0.004**	**0.67**	**0.002**	**0.66**	**0.003**	0.16	0.536
**Sensory nerves**												
Amp tibial (μV)	**–0.62**	**0.008**	**–0.57**	**0.018**	–0.31	0.246	0.34	0.188	0.37	0.141	0.24	0.347
CV tibial (m.s^{-1})	–0.13	0.607	–0.45	0.064	–0.18	0.497	0.21	0.401	0.05	0.844	0.21	0.406
Amp ulnar (μV)	**–0.53**	**0.030**	–0.44	0.076	–0.45	0.069	0.13	0.620	0.10	0.696	0.03	0.914
CV ulnar (m.s^{-1})	–0.24	0.338	–0.01	0.983	–0.31	0.233	0.21	0.409	0.25	0.316	0.30	0.229

Coef: correlation coefficient (*r* for all variables except for the motor amplitude at carpus, sensory amplitude tibial, sensory CV ulnar and all RMTS correlations, where Spearman's ρ was calculated); Amp: amplitude; CV: conduction velocity, Rx: radiographic; MI-CAT(V): Montreal instrument for cat arthritis testing for use by veterinarians; PWT: paw withdrawal threshold; RMTS: response to mechanical temporal summation.
